# Is there a risk of addiction to ketamine during the treatment of depression? A systematic review of available literature

**DOI:** 10.1192/j.eurpsy.2025.466

**Published:** 2025-08-26

**Authors:** G. Ingrosso, A. Cleare, M. Juruena

**Affiliations:** 1 Institute of Psychiatry, Psychology and Neuroscience, King’s College London, Centre for Affective Disorders, London, United Kingdom; 2 Department of Health Sciences, University of Milan, Milan, Italy

## Abstract

**Introduction:**

Depression is a leading cause of disability globally, and while conventional treatments, such as antidepressants and psychological therapy, benefit many patients, a significant proportion fail to achieve symptom relief. Ketamine has demonstrated both rapid and sustained efficacy in treating depression, especially in treatment-resistant cases. However, concerns regarding the addictive potential of ketamine during long-term depression treatment persist among clinicians.

**Objectives:**

The review aimed to assess the prevalence of addiction phenomena associated with ketamine treatment of depression in adult populations.

**Methods:**

The review followed PRISMA guidelines and was pre-registered on PROSPERO (CD42023435468). A comprehensive search was conducted in Medline, Embase, PsycInfo and Global Health databases, with additional relevant studies identified through reference lists. Data extraction and study selection were performed by two independent reviewers. Risk of bias was assessed using appropriate tools based on study design.

**Results:**

Sixteen studies were included, comprising six randomised controlled trials, three single-arm open-label studies, one retrospective study, three case series and three case reports, for a total of 2174 patients. The studies employed various routes of administration, including intravenous, intramuscular, intranasal, oral and sublingual. Ketamine was administered in the racemic form, except for the studies that utilised intranasal esketamine. Four patients were reported to exhibit clear signs of tolerance to the antidepressant effects of ketamine or dependence on the drug, while the majority did not. Additionally, papers discussing addiction phenomena in studies that did not meet the inclusion criteria are also reviewed.

**Conclusions:**

Despite the heterogeneity in study designs and outcome assessment methods, the review underscores the relative safety of ketamine treatment for adult patients with depression, emphasising the importance of medically supervised administration, vigilant monitoring and judicious dosing. Future long-term studies employing quantitative scales to assess dependence phenomena could contribute to strengthen the evidence for a safe and effective use of ketamine in the treatment of depression.

**Disclosure of Interest:**

None Declared

